# Feline Panleukopenia Virus in Dogs from Italy and Egypt

**DOI:** 10.3201/eid2809.220388

**Published:** 2022-09

**Authors:** Georgia Diakoudi, Costantina Desario, Gianvito Lanave, Stefania Salucci, Linda A. Ndiana, Aya Attia Koraney Zarea, Ehab Ali Fouad, Alessio Lorusso, Flora Alfano, Alessandra Cavalli, Canio Buonavoglia, Vito Martella, Nicola Decaro

**Affiliations:** University of Bari Aldo Moro, Bari, Italy (G. Diakoudi, C. Desario, G. Lanave, L.A. Ndiana, A.A. Koraney Zarea, A. Cavalli, C. Buonavoglia, V. Martella, N. Decaro);; Istituto Zooprofilattico Sperimentale dell’Abruzzo e Molise G. Caporale, Teramo, Italy (S. Salucci, A. Lorusso);; National Research Centre, Giza, Egypt (E.A. Fouad);; Istituto Zooprofilattico Sperimentale del Mezzogiorno, Portici, Italy (F. Alfano)

**Keywords:** feline panleukopenia virus, viruses, enteric infections, dogs, parvovirus, sequence analysis, Italy, Egypt

## Abstract

Canine parvovirus and feline panleukopenia virus (FPV) are variants of *Carnivore protoparvovirus 1*. We identified and characterized FPV in dogs from Italy and Egypt using genomic sequencing and phylogenetic analyses. Cost-effective sequencing strategies should be used to monitor interspecies spread, evolution dynamics, and potential host jumping of FPV.

Canine parvovirus (CPV or CPV-2) and feline panleukopenia virus (FPV) are variants of *Carnivore protoparvovirus 1* and major pathogens of domestic and wild carnivores. The linear, single-stranded DNA genome contains 2 open reading frames that encode 2 nonstructural and 2 capsid proteins (*1*).

FPV and CPV are closely related antigenically and genetically (≈98% identity at the nucleotide level) but differ in host range and pathogenicity. These biological differences are determined by amino acid mutations in the VP2 capsid protein (*2*). CPV-2 antigenic variants 2a, 2b, and 2c are able to infect felids and cause FPV-like disease (*2*). FPV is believed to be incapable of infecting dogs but has been shown to replicate in some canine tissues after experimental oronasal infection (*3*). Furthermore, studies have reported the presence of FPV in dogs with CPV-like gastroenteritis (*4*–*8*).

The Infectious Diseases Unit, Department of Veterinary Medicine, University of Bari, Italy, has performed routine screening and characterization of canine samples for CPV and FPV since the mid-1990s and has combined traditional virological and molecular techniques to differentiate between FPV and CPV types 2a/2b and 2b/2c (*9*). During 2019–2021, we screened and typed ≈1,000 *Carnivore protoparvovirus 1* strains from cats and dogs. In 2021, FPV was unexpectedly identified in dogs from epidemiologically unrelated cases. We identified FPV in a blood sample obtained from a 1-year-old dog from Giza, Egypt, that had an unexplained fever (case A) and in fecal samples from 3 dead adult dogs in Teramo, Italy (case series B). The dogs from Italy had severe gastrointestinal symptoms that the attending veterinarian initially suspected were from poisoning.

Because finding FPV in dogs is unusual, we analyzed the samples by using 2 PCR primer sets that differentiated between canine and feline cytochrome b genes (*10*). We confirmed that the FPV samples were of canine origin. Moreover, toxicologic analysis of the 3 dogs in case series B excluded a diagnosis of poisoning, and other enteric canine pathogens were excluded as causes of the gastrointestinal symptoms by using culture and molecular assays. We performed immunofluorescence analyses of tissue from the small intestines of the 3 dogs in case series B and detected parvoviral antigens in epithelial cells (Appendix Figure 1). Attempts to isolate the virus by infecting feline or canine cell lines were unsuccessful, likely because of low viral titers.

To acquire complete viral genome sequences of samples from each case, we performed genomic PCR by using LA Taq polymerase (Takara Bio, http://www.takarabio.com). PCR products were used for library preparation. We performed adaptor ligation of genomic DNA by using the Ligation Sequencing Kit (Oxford Nanopore Technologies, https://nanoporetech.com) according to the manufacturer’s guidelines. Sequencing was performed by using the FLO-MIN106D Flow Cell, R9 version, and MinION Mk1C sequencing platform (Oxford Nanopore Technologies).

We obtained complete sequences of coding regions for the virus strains from Italy (ΙΤΑ/2021/164-1; GenBank accession no. OM638042) and Egypt (EGY/2021/139-188; GenBank accession no. OM638043). The ΙΤΑ/2021/164-1 and EGY/2021/139-188 strains were characterized as FPV on the basis of sequence and phylogenetic analyses. We aligned amino acid sequences for VP2 from FPV, CPV, and the dog-associated FPV strains from Italy and Egypt to determine biological differences between the variants (Figure). We identified an I101T aa substitution in our cases that is likely associated with host range determination (Figure) (*2*). The FPV strains from Italy and Egypt segregated into different phylogenetic clusters (Appendix Figure 2).

**Figure:**
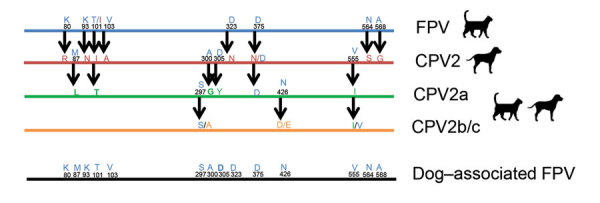
Genetic differences in feline panleukopenia virus in dogs from Italy and Egypt. Amino acid residues in the capsid protein VP2 differed between FPV, CPV-2, CPV-2a, CPV-2b, and CPV-2c variants of *Carnivore protoparvovirus 1*. Colors indicate variant origins of amino acid residues. We identified an I101T aa substitution mutation in FPV from these dog-associated cases. CPV, canine parvovirus; FPV, feline panleukopenia virus.

FPV has been recently reported in Pakistan, Vietnam, Thailand, and China in dogs that had gastroenteritis (*4*–*8*). These viruses were characterized as FPV after partial or complete sequence analysis of the gene encoding VP2. A unique K93N substitution mutation involved in host range control (*2*) was identified in an FPV strain in Thailand (*4*), and I101T mutations were found in dog-associated FPV strains from Vietnam (*5*) and China (*6*). As noted, the I101T mutation was also found in our dog-associated FPV strains. I101 has been observed in prototypical FPV strains, whereas T101 has been found in recent FPV isolates (*5*). The I101T substitution has also been observed in CPV-2 and its variant CPV-2a and is believed to be a further adaptation of CPV to the canine host (*2*).

In conclusion, we identified FPV from unrelated cases in dogs. In case A, fever was the only clinical sign in a young dog, whereas a fatal systemic syndrome with enteric signs occurred in 3 adult dogs in case series B. However, the role of FPV in these cases remains unclear. Adoption of cost-effective sequencing strategies in recent years has demonstrated that residual circulation of FPV or FPV-like viruses occurs in dogs in some settings. Genomic sequencing and further phylogenetic analyses can be used to monitor the spread, evolution, and potential host jumping of *Carnivore protoparvovirus 1* variants in domestic and wild carnivores.

Appendix Additional information for feline panleukopenia virus in dogs from Italy and Egypt.
