# Supplementary material for: Feline Panleukopenia Virus in Dogs from Italy and Egypt
Source: Emerg Infect Dis. 2022 Sep;28(9):1933–5. doi: 10.3201/eid2809.220388 (PMC9423925; doi:10.3201/eid2809.220388)
Supplement: Appendix — Additional information for feline panleukopenia virus in dogs from Italy and Egypt. [file 22-0388-Techapp-s1.pdf]

Article DOI: https://doi.org.10.3201/eid2809.220388

Feline Panleukopenia Virus in Dogs from Italy and Egypt

Appendix

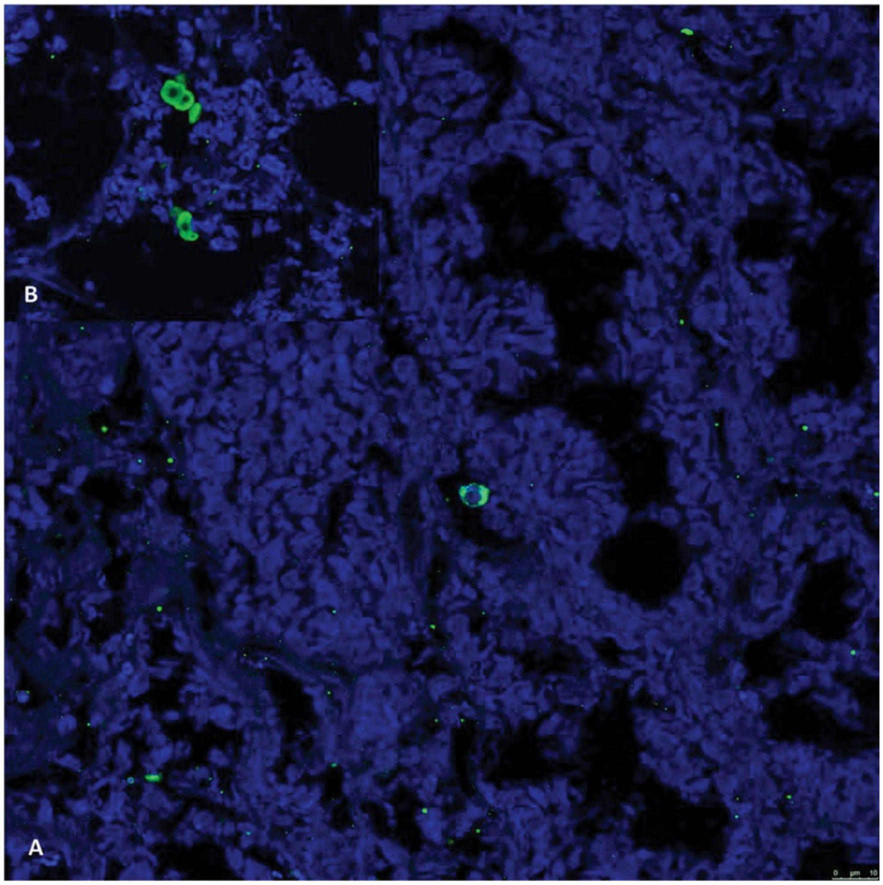

Appendix Figure 1. Immunofluorescence analyses of feline panleukopenia virus in small intestines of dogs from Italy. We prepared 3 μm-thick sections of formalin-fixed, paraffin-embedded samples of small intestine from 3 adult dogs in Italy (case series B) for immunofluorescence analyses. The dogs had severe gastrointestinal symptoms and died. Canine parvovirus (CPV) and feline panleukopenia virus are antigenically similar variants of *Carnivore protoparvovirus 1*. Therefore, we used an anti-CPV antibody (1:50 dilution, rabbit polyclonal antibody) and a fluorescein isothiocyanate-conjugated goat anti-rabbit IgG secondary antibody (1:100, ThermoFisher Scientific, https://www.thermofisher.com) to detect *Carnivore protoparvovirus 1* antigens. A) and B) Representative images of parvoviral antigens detected in epithelial cells of atrophic villi of the small intestine of the 3 dogs; control sections of dog intestinal tissues were negative. Scale bar indicates 10 μm.

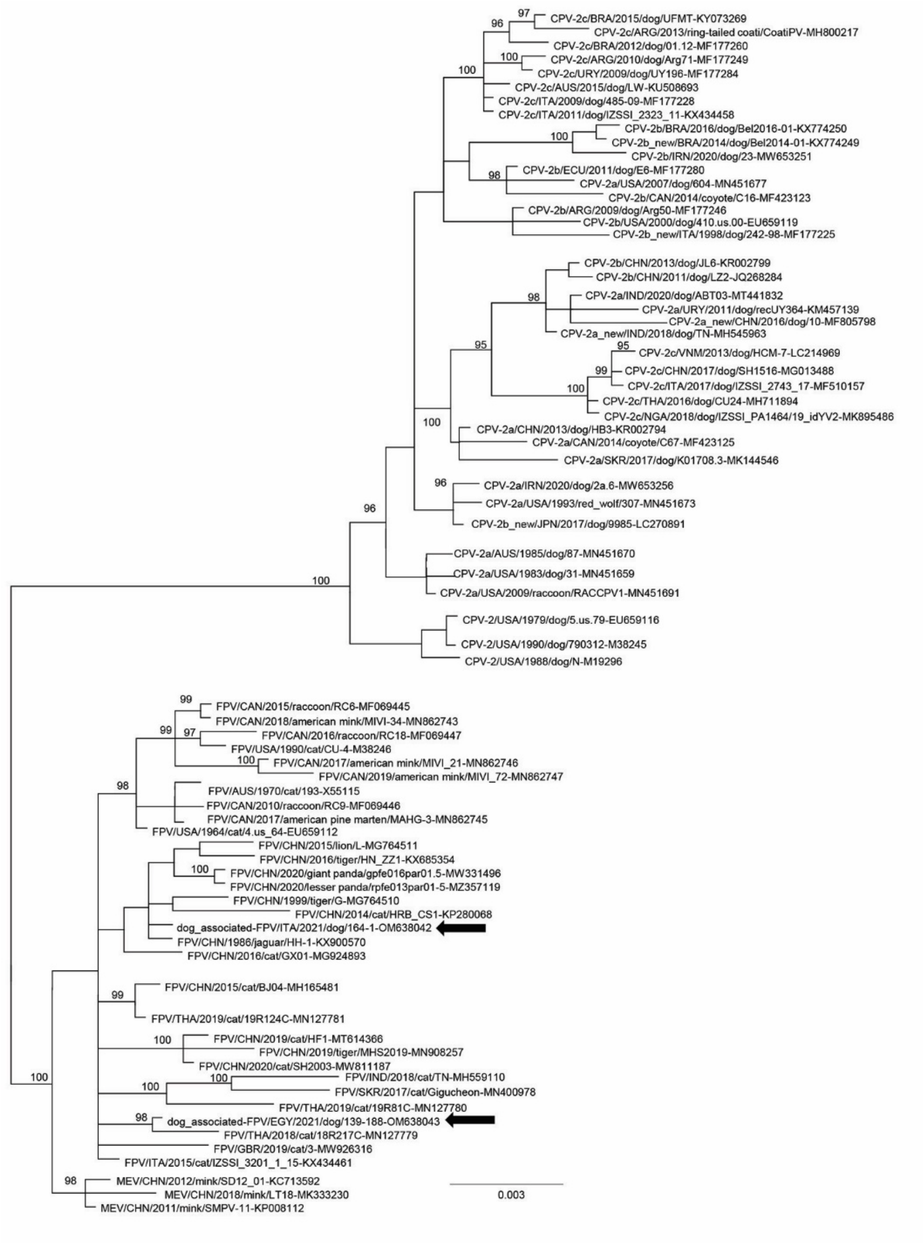

Appendix Figure 2. Phylogenetic differences for feline panleukopenia virus in dogs from Italy and Egypt. Bayesian phylogenetic tree based on ORF2 of *Carnivore protoparvovirus 1*. We aligned a 1,755 nucleotide sequence of ORF2 from the dog-associated FPV strains identified in this study with cognate sequences of *Carnivore protoparvovirus 1* strains retrieved from GenBank. Posterior output for the tree was derived by using a general time-reversible model, proportion of invariable sites, gamma distribution of rate variation across sites, and subsampling frequency of 1,000. Posterior probability values >95% are indicated at the tree nodes. Black arrows indicate the dog-associated FPV strains ITA/2021/164–1 (GenBank accession no. OM638042) and EGY/2021/139–188 (GenBank accession no. OM638043) identified in this study. Scale bar indicates nucleotide substitutions per site. CPV, canine parvovirus; FPV, feline panleukopenia virus; ORF2, open reading frame 2.
